# Dendrimers Containing Ferrocene and Porphyrin Moieties: Synthesis and Cubic Non-Linear Optical Behavior

**DOI:** 10.3390/molecules15042564

**Published:** 2010-04-12

**Authors:** Eric G. Morales-Espinoza, Karla E. Sanchez-Montes, Elena Klimova, Tatiana Klimova, Irina V. Lijanova, José L. Maldonado, Gabriel Ramos-Ortíz, Simón Hernández-Ortega, Marcos Martínez-García

**Affiliations:** 1 Instituto de Química, Universidad Nacional Autónoma de México, Cd. Universitaria, Circuito Exterior, Coyoacán, C.P. 04510, México D.F., Mexico; 2 Facultad de Química, Universidad Nacional Autónoma de México, Cd. Universitaria, Circuito Interior, Coyoacán, C.P. 04510, México D.F., Mexico; 3 Instituto Politécnico Nacional, CIITEC, Cerrada Cecati S/N, Colonia Santa Catarina de Azcapotzalco, C.P. 02250, México D.F., Mexico; 4 Centro de Investigaciones en Óptica, A.P. 1-948, C.P. 37000 León, Gto., Mexico

**Keywords:** ferrocene, porphyrin, dendrimers, non-linear optics

## Abstract

Dendrons with ferrocenyl ended groups joined by styryl moieties were attached to a porphyrin core. All the dendrons used for dendrimer synthesis showed *trans *configuration. The chemical structure of the first generation dendron was confirmed by X-ray crystallographic studies. The structure of the synthesized dendrimers was confirmed by ^1^H- and ^13^C-NMR, electrospray mass spectrometry and elemental analysis. Cubic non-linear optical behavior of the ferrocene and porphyrin-containing dendrimers was studied in solid thin films by THG Maker-Fringe technique at 1,260 nm.

## 1. Introduction

Dendrimers are highly symmetric molecules and possess well-defined nanostructures [1,2,3,4]. A great variety of functional units can be incorporated on the exterior surface or in the interior of these nanostructures [5,6,7], allowing by this way to control the microenvironment inside and around the dendrimers. This property has been extensively explored for different applications. For example, in shape-selective catalysis [8,9], solubilization or protection of molecules [10,11], non-linear optics [12,13], or measuring oxygen content [14,15,16,17]. Dendrimers with redox-active moieties [18,19,20] such as ferrocene introduced in the structure [21] are of great interest as single-molecule electron pools (molecular batteries) [22], hosts for anion recognition [23,24], and electrochemical biosensors [25]. Dendrimers in which a photoactive group is present along with multiple redox-active moieties can find particularly interesting applications. Indeed, there are reports of dendrimers that have a photoactive group (e.g., porphyrin) at the core and multiple redox-active groups attached to the dendritic framework [25]. The non-linear optical (NLO) properties of porphyrins and metal-substituted porphyrins have been extensively studied [26,27]. Furthermore, recently the NLO features of several dendrimers have been reported [28,29,30,31,32,33,34,35,36,37]. The use of novel nanostructured “metal-containing dendrimers for electronic and optical applications is a very important issue for the creation of new devices [28,30,32,33,36]. These compounds belong to the family of organometallic materials which could possess strong π-electron conjugation, *i.e.* extended electron delocalization through the molecule. Since this is a crucial factor in attaining high optical non-linearities, it is of great interest to identify and understand the structure-property relationship of these compounds. This knowledge will contribute to a rational design of new third-order NLO [26,27,31,32,33,37] materials based on low molecular weight molecules, macromolecules and polymers. In this paper, we report the synthesis of monodisperse architectural isomers of poly(ferrocenylstyryl) dendrons and dendrimers with a porphyrin core. Furthermore, cubic NLO behavior is reported for such dendrimers containing in the molecule both ferrocene and porphyrin units.

## 2. Results and Discussion

**Scheme 1:**
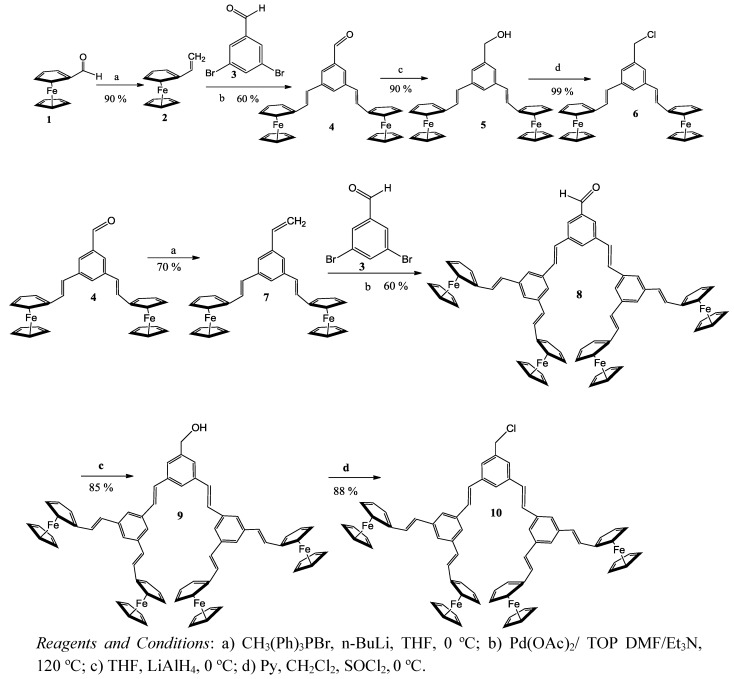
Synthesis of first and second generation dendrons.

Dendrons containing ferrocenyl groups were prepared according to the convergent Fréchet approach [38]. Vinyl ferrocene was synthesized from ferrocene carboxaldehyde by a Wittig reaction (Scheme 1). Subsequent Heck reaction coupling of the vinyl ferrocene **2** and 3,5-dibromo-benzaldehyde (**3**) in dimethylformamide and triethylamine using palladium acetate as catalyst afforded **4**. This was reduced with LiAlH_4_ in THF at 0 ºC to give alcohol **5**, which was converted into the chloride **6** upon treatment with thionyl chloride in dichloromethane at 0 ºC. The chloride **6** was used as the reagent for the synthesis of the first generation of ferrocenyl-containing dendrimers [39]. Chloride **10, **a second generation dendron, was obtained following the same methodology (Scheme 1). The ^1^H-NMR spectral data showed that all the dendrons had *E* stereochemistry of the double bonds in them [39].

**Figure 1:**
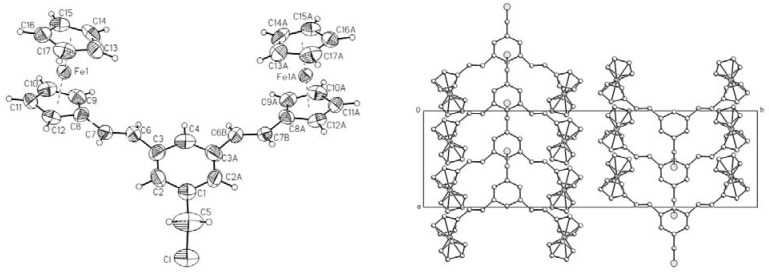
Crystal structure and crystal packing of dendron **6**. Selected bond lengths (Å): C(4)-C(3) = 1.381(5), C(3)-C(6) = 1.525(5), C(6)-C(7) = 1.310(7), C(7)-C(8) = 1.522(5), C(8)-C(9) = 1.407(5). Selected bond angles (^o^): C(7)-C(6)-C(3) = 116.8(5), C(6)-C(7)-C(8) = 119.8(6), C(7A)-C(6A)-C(3) = 119.1(5), C(6A)-C(7A)-C(8) = 118.5(6).

The structure of compound **6** was also determined by X-ray diffraction analysis of single crystal prepared by crystallization from chloroform. The general view of dendron **6 **is shown in Figure 1. 

**Scheme 2:**
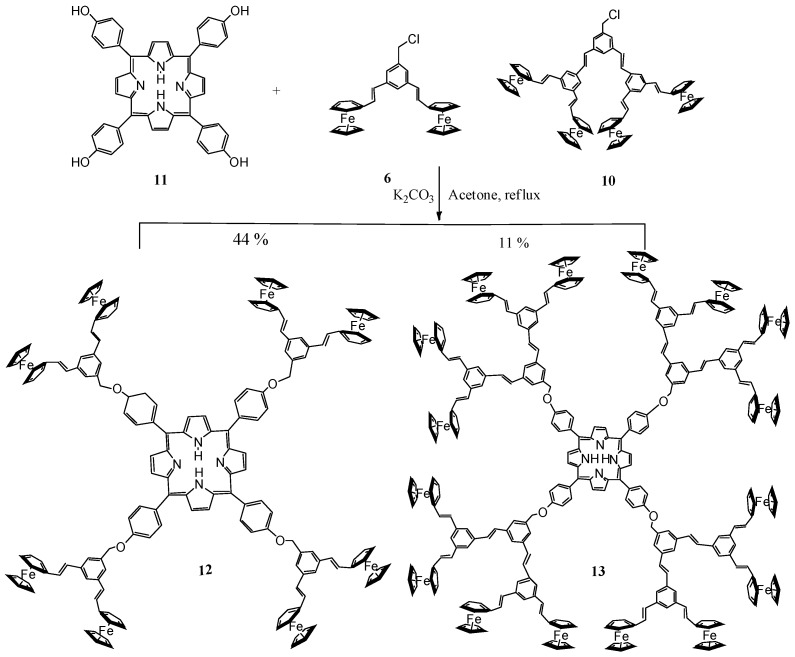
Synthesis of the dendrimers containing a porphyrin core and ferrocene units in the periphery.

The dendrimers were obtained in one step by an *O*-alkylation between dendrons **6 **or **10** and porphyrin **11** (Scheme 2).

**Figure 2:**
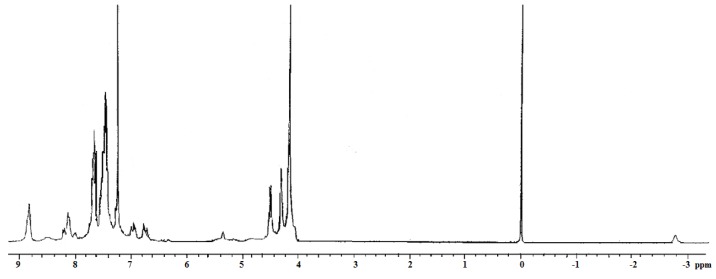
^1^H-NMR spectrum of the second generation dendrimer **12** in CDCl_3_ at room temperature.

The reaction was carried out in acetone and K_2_CO_3_ at reflux for 7 days and the dendrimers were obtained in good yields. In the ^1^H-NMR spectrum of dendrimer **12 **(Figure 2) the following signals were observed: one broad signal at δ_Η_-2.76 due the protons inside the porphyrin ring, the characteristic signals at δ_Η __-_4.16, at δ_Η _4.31 and at δ_Η_4.50 due to the ferrocenyl groups, one singlet at δ_Η _5.35 due to the CH_2_-O, two doublets at δ_Η_6.76, and δ_Η _6.98 due to the CH= groups with a coupling constant *J *= 16.2 and 15.6 Hz and the signals at δ_Η_7.41–8.21 due to the aromatic protons. Finally, one singlet was observed at δ_Η_8.85 due to the protons at the pyrrole ring. 

### 2.1. Linear and third order non-linear optical characterization

**Figure 3:**
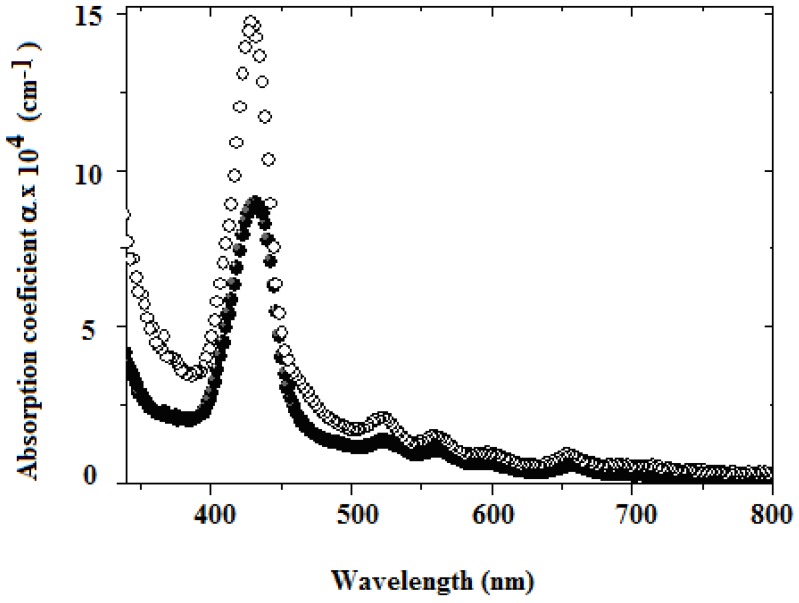
Optical linear absorption coefficient of polymer films doped ferrocene and porphyrin-containing dendrimers **12** (filled circles) and **13 **(open circles).

Figure 3 shows the linear absorption coefficient of the compounds **12** and **13** doped into solid polystyrene (PS) films at a loading level of 50 wt. %. Sample thickness was between 50 nm and 180 nm. The films showed a broad absorption band with a main maximum about 430 nm. There are secondary maxima at 524, 560, 598, and 656 nm. These absorption spectra are not corrected by Fresnel losses at the interfaces of the film and substrate. 

In the present work, the cubic NLO response for the dendrimers **12** and **13** containing ferrocene and porphyrin groups was estimated by the use of third-harmonic generation (THG) Maker-fringes technique [40]. This technique was selected to determine χ^(3)^ because it allows measuring pure electronic NLO effects, which is important for high bandwidth photonic applications. 

**Figure 4:**
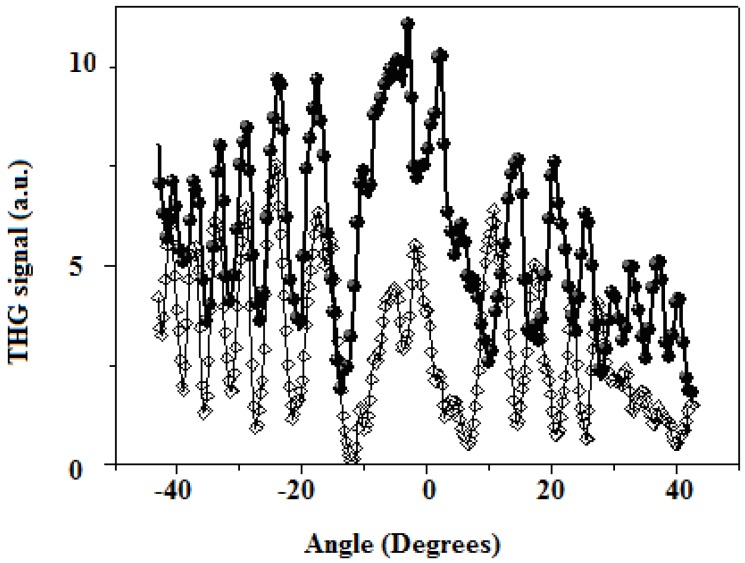
THG Maker-fringe pattern for 174 nm-thin polymer film doped with 50 wt. % of compound **13** (filled circles) and for a 1-mm-thick substrate without a film deposited on it (open diamonds); lines are guides for the eye. The fundamental wavelength was 1260 nm.

Figure 4 shows the THG Maker-Fringe pattern for compound **13** doped into PS film (sample thickness: 174 nm). As reference, the figure also includes the THG pattern measured from the fused silica substrate alone (thickness: 1 mm). These data were obtained at the fundamental near infrared wavelength of 1,260 nm (THG signal at 420 nm). From these data, it was estimated that the third-order non-linear susceptibility of the polymer film doped with compound **13 **is of the order of 8.3 × 10^-12^ esu Table 1 shows the χ^(3)^ values for the compounds studied. The absorption coefficient α at 420 nm was taken into account. 

From our measurements, it is clear that for these compounds the cubic susceptibility χ^(3)^ is improved for ferrocene and porphyrin-containing dendrimer **13 **in comparison with dendrimer**12, **even considering the slight difference in the absorption coefficient α: 7.1 and 11.4 × 10^5^ (cm^-1^) for **12** and **13**, respectively, at 420 nm. This could be due to the presence of more ferrocenyl groups in the structure of dendrimer **13**. Reports of third-order NLO-properties of similar compounds are limited. Typical techniques used to measure cubic non-linearities include Z-scan, Degenerate Four-Wave Mixing (DFWM) and THG. These differences in characterization techniques, as well as the variety of wavelengths employed and the fact that most of the non-linear characterization is performed with solutions, makes the comparison between the optical non-linearities reported for similar molecules not straightforward. Previously, χ^(3)^ values of the order of 10^-10^-10^-12^ esu were reported for some other dendrimers [33,35]. However, in these works [33,35] different NLO techniques such as DFWM [33], Z-scan and self-phase modulation [35] were used. Also, in the reference [35], samples were tested in solution and a femtosecond laser system was used for the excitation. For some particular dendrimers with CdS quantum dots, χ^(3)^ of the order of 10^-9^ esu was obtained by Z-scan technique with a picosecond laser system [37]. In our previous report [39] on resorcinarene-based dendrimers with phenyl and ferrocenyl-ended groups, cubic susceptibilities were of the order of 5 × 10^-13^ to 2 × 10^-12^ esu.

**Table 1:** χ^(3)^ Values. Fundamental wavelength: 1260 nm.

Sample (50 wt. % into PS)	α × 10^5^ (cm^-1^)^a)^	(× 10^-12^ esu)^b)^
**12**	7.1	3.1
**13**	11.4	8.3

^a)^ At 420 nm (THG of 1260 nm) ^b)^ χ^(3)^ for fused silica = 3.1 × 10^-14^ esu.

### 2.2. Crystal structure determination

A suitable crystal of compound **6 **(obtained by crystallization from CH_2_Cl_2 _at room temperature) was rolled in epoxy resin and mounted on a glass fiber. Bruker Apex AXS CCD area detector X-Ray diffractometer was the instrument used for the determination. The data were first reduced and corrected for absorption using psi-scans, and then solved using the program SHELL-XS. All nonhydrogen atoms were refined with anisotropic thermal parameters and the hydrogen atoms were refined at calculated positions with thermal parameters constrained to the carbon atom on which they were attached. A summary of the key crystallographic information is given in Table 2. CCDC 764775 contains the supplementary crystallographic data for this paper. These data can be obtained free of charge via www.ccdc.cam.ac.uk/conts/retrieving.html (or from the CCDC, 12 Union Road, Cambridge CB2 1EZ, UK; fax: +44 1223 336033; e-mail: deposit@ccdc.cam.ac.uk)

**Table 2:** Crystal data and structure refinement.

Empirical formula	C_31_ H_27_ Cl Fe_2_
Formula weight	546.68
Temperature	298(2) K
Wavelength	0.71073 Å
Crystal system	Orthorhombic
Space group	Pnma
Unit cell dimensions	a = 8.8302(7) Å
	b = 30.601(2) Å
	c = 9.349(1) Å
Volume	2526.2(4) Å3
Z	4
Density (calculated)	1.437 Mg/m3
Absorption coefficient	1.272 mm-1
F(000)	1128
Crystal size / shape / color	0.26 × 0.24 × 0.09 mm / Prism/ Red
Theta range for data collection	2.28 to 25.36°.
Index ranges	-10<= h <=10, -36<= k <=36, -11<= l <=11
Reflections collected	19597
Independent reflections	2362 [R(int) = 0.0404]
Completeness to theta = 25.36°	99.8 %
Absorption correction	Integration
Max. and min. transmission	0.8941 and 0.7334
Refinement method	Full-matrix least-squares on F2
Data / restraints / parameters	2362/60/179
Goodness-of-fit on F2	1.090
Final R indices [I>2sigma(I)]	R1 = 0.0465, wR2 = 0.1059
R indices (all data)	R1 = 0.0591, wR2 = 0.1130
Largest diff. peak and hole	0.429 and -0.310 e. Å-3

## 3. Experimental

### 3.1. General

Solvents and reagents were purchased as reagent grade and used without further purification. Acetone was distilled over calcium chloride. Tetrahydrofuran was distilled from sodium and benzophenone. Column chromatography was performed on Merck silica gel 60Å (70-230 mesh). ^1^H- and ^13^C-NMR were recorded on a Varian-Unity-300 MHz with tetramethylsilane (TMS) as an internal reference. Infrared (IR) spectra were measured on a spectrophotometer Nicolet FT-SSX. Elemental analysis was determined by Galbraith Laboratories Inc. (Knoxville, TN, USA). FAB+ mass spectra were taken on a JEOL JMS AX505 HA instrument. Electrospray mass spectra were taken on a Bruker Daltonic, Esquire 6000. MALDI-TOF mass spectra were taken on a Bruker Omni FLEX. 

### 3.2. Synthesis of dendrons and dendrimers

Compounds **2-10** were obtained following the previously reported methodology [39]. A mixture of of the respective dendron **6** or **10 **(1 mmol), potassium carbonate (21.2 mmol) and 18-crown-6 (0.56 g, 2.12 mmol) in dry acetone (80 mL) was heated to reflux and stirred vigorously under a nitrogen atmosphere for 20 min. The compound **11 **(0.0125 mmol) dissolved in dry acetone (40 mL) was added dropwise, and the reaction was continued for 7 days. The mixture was allowed to cool and the precipitate was filtered. The filtrate was evaporated to dryness under reduced pressure. The residue dissolved in diethyl ether was washed with an aqueous solution of 5% Na_2_CO_3_ (3 times). The organic layer was dried and evaporated to dryness and the dendrimers were purified using the following procedure: the dendrimer was dissolved in CH_2_Cl_2_, then methanol was added producing the precipitation of the dendrimer back. This procedure was repeated three times. 

*Dendrimer ***12**. Yield 0.24 g (44%), black powder, m.p. > 300 ºC. UV-Vis CH_2_Cl_2_ (nm): 693, 651, 593, 555, 519, 455, 421, 312, 262, 231. Absorption coefficient α: 7.1 × 10^5^ (cm^-1^). IR (KBr, cm^-1^): 3093, 2925, 1599, 1505, 1238, 1175, 958, 806, 753. ^1^H-NMR (300 MHz, CDCl_3_), δ_H_ (ppm): -2.72 (s, 2H, N-H), 4.18 (s, 40H, C_5_H_5_), 4.32 (s, 16H, C_5_H_4_), 4.53 (s, 16H, C_5_H_4_), 5.34 (s, 8H, CH_2_-O), 6.76 (d, 8H, =CH_2_, *J *= 16.2 Hz), 6.98 (d, 8H, =CH_2_, *J *= 15.6 Hz), 7.41 (s, 8H, Ar), 7.47 (s, 4H, Ar), 7.54 (d, 8H, Ar, *J *= 6.8 Hz), 8.21 (d, 8H, Ar, *J = *8.4 Hz), 8.89 (s, 8H, py). ^13^C-NMR (75 MHz, CDCl_3_), δ_C_ (ppm): 66.9 (C_5_H_4_), 69.1 (C_5_H_4_), 69.2 (C_5_H_5_), 71.0 (Ar-C-O), 83.1 (C*_ipso_*), 113.1 (Ar_porph_), 119.7 (Ar), 123.2 (py), 123.5 (Ar), 125.7 (CH=), 127.7 (CH=), 131.0 (C*_ipso_*), 134.9 (Ar_porph_), 135.6 (C*_ipso_*), 137.7 (C*_ipso_*), 138.7 (Ar_porph_), 158.6 (C*_ipso_*). Electrospray (m/z): 2719. Anal. calcd. for C_168_H_134_Fe_8_N_4_O_4_; C 74.14, H 5.04%. Found: C 74.10, H 5.10%

Dendrimer **13**. Yield 0.12 g (11%), black powder, m.p. >300 ºC. UV-Vis CH_2_Cl_2_ (nm): 649, 594, 556, 423, 313, 245. Absorption coefficient α: 11.4 × 10^5^ (cm^-1^) IR (KBr, cm^-1^): 2924, 1725, 1597, 1505, 1238, 1173, 805, 753.^ 1^H-NMR (300 MHz, CDCl_3_), δ_H_ (ppm): -2.76 (s, 2H, N-H), 4.16 (s, 80H, C_5_H_5_), 4.31 (s, 32H, C_5_H_4_), 4.50 (s, 32H, C_5_H_4_), 5.35 (s, 8H, CH_2_-O), 6.74 (d, 8H, =CH_2_, *J *= 15.6 Hz), 6.76 (d, 16H, =CH_2_, *J *= 15.6 Hz), 6.96 (d, 8H, =CH_2_, *J *= 15.9 Hz ), 6.98 (d, 16H, =CH_2_, *J *= 15.6 Hz), 7.41 (s, 8H, Ar), 7.47 (d, 16H, Ar, *J *= 7.2 Hz), 7.54 (d, 8H, Ar, *J *= 6.8 Hz), 7.68 (q, 12H, Ar), 8.13 (d, 8H, *J *= 7.2 Hz, Ar), 8.85 (s, 8H, py). ^13^C-NMR (75 MHz, CDCl_3_), δ_C_ (ppm): 64.95 (C_5_H_4_), 66.96 (C_5_H_4_), 69.28 (C_5_H_5_), 73.42 (Ar-C-O), 83.16 (C*_ipso_*), 112.8 (Ar_porph_), 119.68 (C*_ipso_*), 122.52 (Ar_porph_), 125.64 (py), 127.74 (C*_ipso_*), 128.42 (CH=), 128.54 (CH=), 130.92 (Ar), 131.95 (Ar), 132.05 (Ar), 132.18 (Ar), 133.23 (C*_ipso_*), 135.30 (C*_ipso_*), 135.69 (C*_ipso_*), 138.72 (Ar). Electrospray (m/z): 5215. Anal. calcd. for C_328_H_262_Fe_16_N_4_O_4_, C 75.51, H 5.06%. Found: C 75.54, H 5.09%.

### 3.3. THG Maker fringe measurements

The non-linear optical measurements were performed in solid state (solid films) using the guest (molecule)-host (polymer) approach. Mixtures of polystyrene (PS) and dendrimer-porphyrin 50:50 wt % ratio, respectively, were dissolved in dichloromethane. The solid films were deposited on fused silica substrates (1 mm-thick) by using the spin coating technique. The prepared films had typical thickness between 50 and 180 nm with good optical quality. Absorption spectra of spin-coated films were obtained with a spectrophotometer (Perkin-Elmer Lambda 900). Sample thickness was measured by a Dektak 6M profiler. 

The THG Maker-fringes setup is reported elsewhere [41,42]. Briefly, it consisted of a Nd-YAG laser-pumped optical parametric oscillator (OPO) that delivered pulses of 8 ns at a repetition rate of 10 Hz. A fundamental wavelength of 1260 nm (idler beam) was used. The output of the OPO system was focused into the films with a 30-cm focal-length lens to form a spot with a radius of approximately 150 μm. Typical energies in our measurements were set at 1 mJ per pulse at sample position (corresponding to peak intensities of ~0.18 GW/cm^2^). The third-harmonic beam, as a bulk effect, emerging from the films was separated from the pump beam by using a colour filter and detected with a PMT and a Lock-in amplifier. The THG measurements were performed for incident angles in the range from -40° to 40° with steps of 0.27 degrees. All the experiment was computer-controlled.

In the Maker-fringes technique, the third-harmonic peak intensity *I*^3*ω*^ from the substrate-film structure is compared to the one produced from the substrate alone. Then, the non-linear susceptibility χ^(3)^ in a film of thickness *L_f_* is determined from:

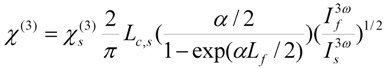
(1)
where *χ*_*S*_^(3)^ and *L_C,S_* are the non-linear susceptibility and coherence length, respectively, for the substrate at the fundamental wavelength, and α is the film absorption coefficient at the harmonic wavelength [43]. In our calculation we considered *χ*_*S*_^(3)^ = 3.1 × 10^-14^ esu and *L_C,S_* = 9.8 *μm* for the fused silica substrate [41,42]. Our samples satisfied the condition *L_f_* << *L_C,S_* in which the Equation (1) is valid.

## 4. Conclusions

Dendrimers **12** and **13** containing ferrocene and porphyrin-groups in the same molecule were synthesized. These dendrimers had 8 and 16 ferrocenyl ended groups joined by styryl moieties to a porphyrin core. All the dendrons used for the synthesis of dendrimers showed *trans *configuration. The chemical structure of dendron **6** was confirmed by X-ray crystallography. Cubic non-linear optical behavior of these first and second generations of dendrimers was studied. The χ^(3)^ values estimated from the THG Maker-Fringe technique were of 3.1 and 8.3 × 10^-12^ esu for **12** and **13**, respectively. Higher cubic susceptibility of **13**, about a factor of 2.7 in comparison with **12**, is due probably to an increase in the number of ferrocenyl groups in the molecule.
